# Repertoire of SSRs in the Castor Bean Genome and Their Utilization in Genetic Diversity Analysis in *Jatropha curcas*

**DOI:** 10.1155/2011/286089

**Published:** 2011-05-22

**Authors:** Arti Sharma, Rajinder Singh Chauhan

**Affiliations:** Department of Biotechnology & Bioinformatics, Jaypee University of Information Technology, Waknaghat, P.O. Dumehar Bani, Kandaghat, Solan 173 215, India

## Abstract

Castor bean and *Jatropha* contain seed oil of industrial importance, share taxonomical and biochemical similarities, which can be explored for identifying SSRs in the whole genome sequence of castor bean and utilized in *Jatropha curcas*. Whole genome analysis of castor bean identified 5,80,986 SSRs with a frequency of 1 per 680 bp. Genomic distribution of SSRs revealed that 27% were present in the non-genic region whereas 73% were also present in the putative genic regions with 26% in 5′UTRs, 25% in introns, 16% in 3′UTRs and 6% in the exons. Dinucleotide repeats were more frequent in introns, 5′UTRs and 3′UTRs whereas trinucleotide repeats were predominant in the exons. The transferability of randomly selected 302 SSRs, from castor bean to 49 *J. curcas* genotypes and 8 *Jatropha* species other than *J. curcas*, showed that 211 (*∼*70%) amplified on *Jatropha* out of which 7.58% showed polymorphisms in *J. curcas* genotypes and 12.32% in *Jatropha* species. The higher rate of transferability of SSR markers from castor bean to *Jatropha* coupled with a good level of PIC (polymorphic information content) value (0.2 in *J. curcas* genotypes and 0.6 in *Jatropha* species) suggested that SSRs would be useful in germplasm analysis, linkage mapping, diversity studies and phylogenetic relationships, and so forth, in *J. curcas* as well as other *Jatropha* species.

## 1. Introduction

Biofuel is a renewable fuel which can be used as an alternative to or an addition to fossil-derived fuels with multitudes of environmental benefits. Off late, various oil seed plants suited to wide agroclimatic conditions have been explored as sources of future fuels due to the fear that the fossil fuels may get exhausted, in addition to their environmental concerns. *Jatropha curcas* is a promising bioenergy crop with more than 35% oil content in its seeds with chemical characteristics of oil suitable to be used in modern combustion engines. The plant species is native to tropical America with a heterozygous genome [1–4]. The taxonomic studies of the genus *Jatropha* have shown that the *J. curcas* is a primitive ancestral species due to its morphological distinctness and other *Jatropha* species evolved from *J. curcas* with changes in growth habit [5]. *J. curcas* crosses readily with other Jatropha species forming natural hybrid complexes (*J. curcas-gossypifolia*). 

The narrow genetic base in crop plants has been a major limitation in their genetic improvement for desirable traits [6, 7]. Previous studies based on RAPD, SSR, and AFLP analysis have indicated that the genetic base of *J. curcas* is narrow [8–11]. Basha et al. [11] demonstrated polymorphisms of 61.8 and 35.5% with RAPDs and ISSRs, respectively. They identified 12 microsatellite primers differentiating the toxic and non-toxic Mexican accessions. Sudheer Pamidimarri et al. [12] identified RAPD, AFLP, and one SSR marker differentiating toxic and non-toxic varieties of *J. curcas*. The *J. curcas* lacks basic genome resources such as genetic map, molecular markers, and genome libraries, thereby necessitating the development of additional molecular markers so as to accelerate the process of genetic improvement programmes. The recent sequencing of *J. curcas* genome will enable further progress in its genomics [13]. 

SSRs occur as frequently as 1 in every 6 kb in the plant genomes [14]. The functional role of SSRs vary with their location in the genome [14, 15]. Variations in SSRs in 5′UTRs and 3′UTRs are known to effect gene expression [16]. For example, SSRs in the 5′UTRs affect gene regulation and/or gene transcription, and SSRs in the 3′UTRs may cause transcription slippage [14, 15]. Large numbers of SSRs have been detected and documented in the transcribed regions of genomes [17, 18] with their usage as genetic markers for genotyping, mapping, and positional cloning of genes in different plant species [19–22]. 

Conservation in structure and function of genetic loci has been documented and utilized in the development of anchor markers such as in grass genomes [23], crucifers [24], and solanaceous plants [25]. The availability of public genome sequence databases provides an easier alternative for the identification of anchor markers, including SSRs using bioinformatics, thereby reducing cost and time span for their development [26–28]. Wen et al. [29] identified 241 EST-SSRs and genomic SSR markers in cassava and demonstrated their transfer and polymorphisms among *J. curcas* accessions. 

The castor bean (*Ricinus communis*) is a perennial shrub with 50–55% seed oil and mainly cultivated in tropical and subtropical areas of India, China, and Brazil. It is taxonomically and biochemically related to *J. curcas* because both belong to Euphorbiaceae. The high level of synteny can, therefore, be expected between both plant species, which can be exploited to develop anchor markers. Genome sequence of castor bean was surveyed for SSRs and utilized in *J. curcas* and other *Jatropha* species [30]. The extent of polymorphisms, in SSRs from putative genic (5′and 3′ UTRs, exons, introns) versus nongenic genome regions and SSRs of different types and repeat motif numbers, was investigated. 

## 2. Materials and Methods

### 2.1. Annotation of Castor Bean Genome for SSRs

The castor bean genome sequence (~400 Mb), consisting of 25,828 contigs (4X coverage), was downloaded from The JCVI website (http://castorbean.tigr.org/), and SSRs were identified using an in-house designed Perl script. The Perl script used regular expressions to locate SSR patterns in the FASTA-formatted sequence files and reported sequence contig ID, SSR motif, number of repeats, and sequence coordinates for each SSR. The minimum repeat unit was defined as six for dinucleotides and five for all other higher-order motifs, including tri-, tetra-, penta-, and hexanucleotides. The FASTA-formatted sequence file was allowed to search for all possible combinations of dinucleotide, trinucleotide, tetranucleotide, and pentanucleotide repeats. Castor bean genome sequence contigs harboring SSRs were annotated for putative open reading frames, including 5′UTRs and 3′UTRs, using gene prediction algorithms of FGenesH (http://linux1.softberry.com/berry.phtml?topic=fgenesh&group=programs&subgroup=gfind), because it was cited as the most accurate gene prediction tool [31, 32]. SSR motifs were identified in exons, introns, 3′UTRs, 5′UTRs, and non-genic regions of castor bean and Primers were designed from the sequences flanking each SSR repeat motif by using Primer 3.0 (http://frodo.wi.mit.edu/primer3/). The target amplicon sizes were set as 300–400 bp with optimal annealing temperature of 60°C and the optimal primer length as 20 bp. From the total SSRs identified in the castor bean genome, primer pairs were designed for randomly selected 302 SSRs with a repeat motif of >10 from different genome regions such as 70 from 5′UTRs, 70 from exons, 42 from 3′UTRs, 57 from introns, and 63 from the non-genic regions. 

### 2.2. Plant Material, DNA Extraction, and PCR

The *J. curcas* genotypes were obtained from the National Bureau of Plant Genetic Resources (NBPGR), New Delhi, India (see Table S1 in supplementary material available on line at doi:101155/2011/286089) and *Jatropha* species, other than *J. curcas,* from Dr. k.T. Parthiban of Forest College and Research Institute, Tamil Nadu Agriculture University, Mettupalayam, India. A representative set of 49 genotypes of *J. curcas* and 9 species of *Jatropha*, from different geographical regions of India, was used in diversity analysis. Total genomic DNA was isolated from unfurled leaves according to a modified CTAB-based procedure [33]. The quality of DNA was checked in 1% agarose gels. The PCR reactions were performed in 25 *μ*L reaction volume following thermocycler profiles, that is, 57–55°C (189 markers), 52–54°C (112 markers), and 51°C (1 marker). Each PCR reaction consisted of 30 ng genomic DNA, varying amounts of primer pairs (0.3-0.4 *μ*M), 1.5 mM Mg^2+^, 200 *μ*M dNTPs, and 0.5 units *Taq *DNA polymerase. Amplification programs included 94°C for 5 min, 30 cycles of 94°C for 45 sec, annealing temperature (57–51°C) for 45 sec, 72°C for 2 min, and a final extension of 7 min at 72°C. Ten *μ*L of each PCR product was mixed with 2 *μ*L of 10X gel loading dye (0.2% bromophenol blue, 0.2% xylene cyanol dye, and 30% glycerol in a TA buffer) and electrophoresed in a 4% agarose gel prepared in 0.5X Tris Borate-EDTA (TBE) buffer (0.05 M Tris, 0.05 M boric acid, 1 mM, EDTA pH 8.0). The gel was run at a constant voltage of 80 volts for 1.5 to 2 h, stained with ethidium bromide, and analyzed using the gel documentation system AlphaImager EP (Alpha Innotech Corp., USA).

### 2.3. Statistical Analysis

PowerMarker version 3.25 [34] and Gen-AlEx version 6.1 [35] were used to measure variability at each locus: the observed heterozygosity (HO), the expected heterozygosity (HE), the polymorphism information content (PIC), and the deviation from Hardy-Weinberg equilibrium (HW). Deviations from Hardy-Weinberg (HW) and tests for linkage disequilibrium were evaluated using Fisher's exact tests and sequential Bonferroni corrections. The polymorphism information content (PIC) of each microsatellite locus was determined as described by Weir [36]: PIC = 1 − Σ*P*_*i*_^2^, where *P*_*i*_ is the frequency of the *i*th allele in the genotypes examined. Pairwise similarity matrices were generated by Jaccard's coefficient of similarity [37] by using the SIMQUAL format of NTSYS-pc [38]. The presence or absence of amplicons in the genotypes was scored as 1 or 0, respectively. A dendrogram was constructed by using the unweighted pair group method with arithmetic average (UPGMA) with the SAHN module of NTSYS-pc to show a phenetic representation of genetic relationships as revealed by the similarity coefficient [39].

## 3. Results

 Computational analysis of 25,828 contigs (4X coverage) of castor bean genome identified 5,80,986 SSRs with a frequency of 1 per 680 bp. The location of SSRs in the putative genic (exons, introns, UTRs) and non-genic regions of the castor bean genome was inferred by annotation of 25,828 contigs with FGenesH gene prediction algorithm. A total of 31,221 genes were predicted in the 25,828 contigs of castor bean. 

### 3.1. Occurrence and Distribution of SSRs in the Castor Bean Genome

Abundance of SSRs in different regions of the castor bean genome showed that 73% were located in the putative genic regions and 27% in the non-genic regions. Comparison of SSR densities in the putative genic regions showed that SSRs were more frequent in 5′UTRs (26%) and introns (25%) followed by 3′UTRs (16%) and exons (6%). Analysis of SSRs in the castor bean genome showed that 51% SSRs were dinucleotide repeats, 29% trinucleotide, 12% tetranucleotide, and 8% pentanucleotide repeats. Dinucleotide repeats were more frequent in the non-genic regions (genome), introns, 5′UTRs and 3′UTRs, whereas trinucleotide repeats were more common in the exons. The tetra- and pentanucleotide repeats were randomly distributed. Among dinucleotide repeats, SSRs with (AT)*_n_* repeat motif were common (43%) with a repeat motif ranging from 7 to 48. The frequency of repeat motifs differed in different genomic regions for example (AT)*_n_* and (AG)*_n_* were predominant in 5′UTRs, (TA)*_n_* and (AATA)*_n_* in 3′UTRs, (AT)*_n_* and (TC)*_n_* in introns, and (AT)*_n_* and (TA)*_n_* in the non-genic regions. Analysis of trinucleotide repeats frequencies out of total SSRs indicated their predominance in the order of TCT/GAA/CGC/TTC. The trinucleotide repeats are runs of particular amino acids. The most frequent amino acid runs identified in the castor bean SSRs were serine (TCT)*_n_* (16.5%), glutamate (GAA)*_n_* (13.6%), arginine (CGC)*_n_* (12.3%), and phenylalanine (TTC)*_n_* (9.7%). 

### 3.2. Transferability of SSRs to *Jatropha* and Their Polymorphism Analysis

The transferability of SSRs (cross genera amplification) from castor bean to *Jatropha *(*J. curcas* genotypes and other *Jatropha* species) for 302 randomly selected SSRs (87 from exons, 78 from nongenic regions, 71 from introns and 66 from 5′and 3′UTRs) showed that 273 amplified on castor bean out of which 211 amplified in 43 genotypes of *J. curcas*. The amplification failure in *J. curcas* genotypes, Urli-Kanchan, KcJK5, Hissar local, SKN-Big, and Hansraj, was 2.6%, 2.6%, 4.8%, 6.23%, 7.1%, and 7.6%, respectively, in comparison to 43 genotypes. Six *Jatropha* species produced amplicons with 211 primer pairs, except for *J. mahotwani*, *J. multifida, *and* J. glandulifera,* where the percent failure was 4.74%, 6.23%, and 8.2%, respectively. Ten percent of the SSRs failed to amplify on castor bean DNA, which was attributed to primer mismatches. Out of 211 SSRs with amplification in *Jatropha*, 36.01% were from exons, 21.8% from introns, 16.6% from UTRs, and 25.6% from non-genic regions. Sixteen SSRs from the 5′UTRs (5), non- genic regions (5), introns (3), and exons (3) showed polymorphism in *J. curcas* genotypes (Table 1). The number of alleles per SSR locus ranged from 2 to 6 with a total of 43 alleles ranging in sizes from 200 bp to 600 bp in *J. curcas* genotypes (Figure S1). Twenty six SSRs from 5′&3′UTRs (12), introns (7), 4 nongenic regions (4), and 3 exons (3) were polymorphic on 9 *Jatropha* species (*J. maheshwarii*, *J. multifida*, *J. gossypifolia*, *J. podagrica*, *J. glandulifera*, *J. curcas, J. tanjorensis, J. villosa,*and* J. integerrima*) (Table 2). The number of alleles per SSR locus ranged from 2 to 7 in *Jatropha* species (Figure S2). Five SSRs (JM8, JM10, JM11, JM15, and JM16) showed polymorphisms in *J. curcas* genotypes as well as *Jatropha* species. The transferability of SSRs was the highest to *J. curcas* (~70%) and the lowest to *J. glandulifera* (58%) with a transferability of 63–68% to other species. The level of polymorphism was higher (37.8%) in SSRs from 5′UTRs followed by 24.3% from introns, 18.9% from non-genic regions, 10.8% from exons, and 8.10% from 3′UTRs. The SSRs with dinucleotide repeat motifs showed higher levels of polymorphisms compared to trinucleotide repeats. Out of 37 SSRs, which were polymorphic in *J. curcas* and other *Jatropha* species, 35 (94.5%) were dinucleotide repeats. Tetra- and pentanucleotide repeats did not show any polymorphism in *J. curcas* and other *Jatropha* species.

**Figure 1:**
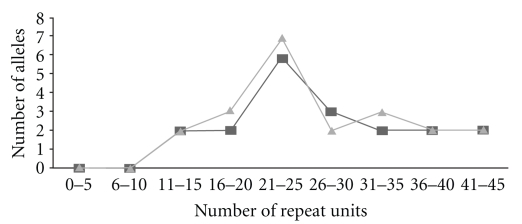
Number of alleles per locus for SSRs of different repeat units in *J. curcas* genotypes (black) and *Jatropha* species (gray).

 Out of all SSRs, which were successfully transferred to *J. curcas* and other *Jatropha* species, 50% contained 15 to 30 repeat units whereas 20% of the SSRs had repeat units of >30. The majority of SSRs with successful amplification and polymorphisms contained more than 15 repeat units (Table 3). The PIC values for polymorphic SSRs in *J. curcas* genotypes and *Jatropha* species varied from 0.1 to 0.5 with an average of 0.2 and 0.3 to 0.7 with an average of 0.5, respectively. The SSRs with dinucleotide repeat motifs showed higher allele numbers (average 2.7 per locus) followed by trinucleotide (average alleles 2.3 per locus). To understand possible relationship between polymorphism of SSR markers with repeat unit length in *J. curcas* genotypes and *Jatropha* species, a line graph was plotted between repeat unit length and numbers of alleles detected (Figure 1). Wide variation in the number of alleles for SSRs with 16 and 25 repeat motifs was seen compared to SSRs with low or high numbers of repeat motifs. An exception to this observation was for SSR, JM15, which contained maximum number of repeat units (TA)_42_ with only two alleles, whereas SSR, JM20 with lower repeat motifs (TA)_23_ showed the highest number of alleles (7). 

### 3.3. Genetic Diversity Analysis among *J. curcas* Genotypes and *Jatropha* Species

The major allele frequency (MAF) ranged from 0.4 to 0.9 for *J. curcas* genotypes and 0.1 to 0.5 for *Jatropha* species. The observed heterozygosity (HO) ranged from 0.1 to 0.5 (mean = 0.2) in *J. curcas* genotypes and 0.4 to 0.7 (mean = 0.6) in *Jatropha* species, and expected (HE) heterozygosities ranged from 0.1 to 0.5 (mean = 0.2) in *J. curcas* genotypes and 0.4 to 0.7 (mean = 0.6) in *Jatropha* species. Hardy-Weinberg probability tests revealed no significant deviations from expected genotype proportions (*P* > .004). There was no evidence of linkage disequilibrium among loci (*P* > .001) after corrections for multiple tests.

**Figure 2:**
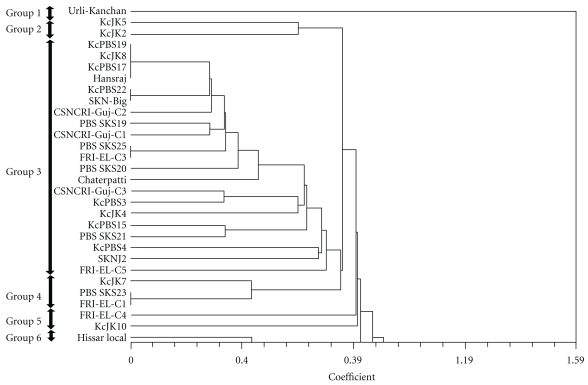
Dendrogram based on allele sharing genetic distances of 49 genotypes of *J. curcas* on the basis of Jaccard's similarity coefficient.

Phylogenetic relationships among different genotypes of *J. curcas* and 9 species of *Jatropha* were inferred based on SSRs analysis. Jaccard's genetic coefficient for *J. curcas* genotypes varied from 1.08 to 9.02. The highest genetic dissimilarity co-efficient (9.02) was observed between 16 polymorphic SSRs in *J. curcas* genotypes while the lowest value (1.08) was measured between eight combinations. The UPGMA cluster analysis of the Jaccard's co-efficient generated a dendrogram for *J. curcas *genotypes, which illustrated the overall genetic relationship among genotypes (Figure 2). Cluster analysis indicated six distinct clusters comprising *J. curcas* genotypes. The *J. curcas* genotype 1 (Urli-Kanchan) and 32 (Hissar local) remained as outliers and formed the first and sixth clusters, respectively. 

## 4. Discussion

Genomic resources of castor bean have been successfully used for the development and utilization of SSR markers in *J. curcas* and other *Jatropha* species, thereby establishing that the SSR markers are a valuable genetic resource for investigating relationships and comparative mapping in Euphorbiaceae. The availability of whole genome sequences and comparative genomics have opened up several avenues for the identification of anchor makers through computational approaches, thus avoiding tedious, costly, and time consuming techniques of genomic or EST library construction for the identification of SSRs. High transferability (70%) of castor bean SSRs to *J. curcas* and other *Jatropha* species shows higher levels of sequence identity between both plant species. The transferability of EST-SSRs (44.63%) and genomic SSRs (29.67%) has been achieved from cassava to *J. curcas* [29]. The high levels of structural and functional synteny has also been observed for other loci between castor bean and *J. curcas* such as for genes involved in the biosynthesis of fatty acids biosynthesis (Sharma & Chauhan, unpublished). The distribution of SSRs in different putative genic regions of the castor bean revealed that SSRs were more prevalent in the 5′UTRs, which was analogous to genomic distributions of SSRs in *A. thaliana*, *B. rapa* and *O. sativa* [18, 40–45]. Contrary to the genomes of *A. thaliana* and *O. sativa *where (AG)*_n_*, (AT)*_n_* and (AC)*_n_* repeats were more abundant, the castor bean genome contained more (AT)*_n_* dinucleotide repeats, which was analogous to *B. rapa* genome [41, 44, 45]. Trinucleotide SSRs were more frequent in the exonic regions of castor bean genome analogous to other genomes. Majority of trinucleotide repeats were in the coding regions of the castor bean genome, which may encode amino acid runs. Frequent occurrence of trinucleotide SSRs in the coding regions has been attributed to their advantages in codon usage whereas the suppression of non-trinucleotide SSRs in the coding regions may be due to the possible risks of their involvement in frameshift mutations [15, 44, 46]. Although biased distribution of codon repeats has been demonstrated in several eukaryotic genomes [15, 41, 44, 46, 47], yet the over-representation of specific amino acid runs varies. The most frequent amino acid runs in *A. thaliana* are serine, proline, glycine, glutamate, glutamine, and aspartate, and those in *O. sativa* are alanine, glycine, proline, serine, arginine, and glutamate [41]. The most frequent amino acid runs in *Brassica rapa* are serine, glutamic acid, aspartic acid, glycine, lysine, and asparagines [45]. Wheras the most frequent amino acid runs in the castor bean SSRs were serine, glutamate, arginine and phenylalanine, which are also the most frequent amino acid runs in the SSRs of other plant genomes [46, 48, 49]. 

It has been reported that SSRs with longer repeat motifs are more informative for detection of polymorphisms [43, 50–54]. For example, Sharma and Chauhan [55] identified an SSR with a long repeat motif (TTC)_31_ in the iron transporter genes of maize showing high polymorphisms among maize inbreds compared to other repeat motifs. On the contrary, we found that the repeat motifs of 16–30 repeat length showed higher polymorphisms than longer repeat motifs of >30 repeat units. Other studies have also found no relationship or weak correlation between SSR polymorphisms and repeat unit length [56–58]. High polymorphisms have been detected in SSRs with dinucleotide repeat motifs in Pearl millet [59] and White clover [60]. 

Overall low level of genetic diversity was detected among *J. curcas* genotypes compared to *Jatropha* species. Basha and Sujatha [9] have reported low levels of diversity among Indian accessions of *J. curcas* indicating a narrow genetic base. Ganesh Ram et al. [1] have shown that polymorphisms with 26 RAPD primers were considerably higher (80.2%) in 8 *Jatropha* species compared to *J. curcas *genotypes. Sun et al. [10] reported one SSR with two alleles and 14.3% polymorphism with 7 AFLP primers in *J. curcas*. 

 The study concludes that the dinucleotide repeat motifs in SSRs, from 5′UTR regions with repeat unit length of 16–30, showed higher polymorphisms suggesting that additional primers can be designed from those SSRs with a higher probability of detecting polymorphisms on castor bean and *J. curcas* and other *Jatropha* species (Table S2). The SSR markers developed in this study would be very useful for germplasm analysis, population genetic structure and taxonomic relationships in *J. curcas *and related taxa.

## Supplementary Material

Supplementary materials contains the list of *J. curcas* genotypes which were used in the present study which were procured from the National Bureau of Plant Genetic Resources (NBPGR), New Delhi, India and a list of additional primer pairs from Castor bean harboring SSRs which can be utilized for transferability and polymorphism survey in Jatropha.

## Figures and Tables

**Table 1:** SSRs used to detect polymorphisms in *Jatropha curcas* genotypes.

SSR locus	Repeat motif	Primer sequence	Location of repeat motif	No. of alleles detected	HO	HE	PIC
JM1	(TA)_22_	F: TTTGAGTGCTCCTATTTGGCTAGAA R: CCAAATGACAAGTAGGCAGAACTTT	5′UTR	3	0.1832	0.1833	0.2
JM2	(TA)_20_	F: GAAATGAGAAGCCTTTACCCTCATT R: AGAGGGAGAAGGGAGAAAGCAGT	5′UTR	2	0.5114	0.5115	0.4
JM3	(AT)_20_	F: TCATCGAATGGTAGAGAACTAATGG R: TTAATTCGGATTCTGAGTCTTGAGG	5′UTR	2	0.2832	0.2832	0.3
JM4	(AT)_29_	F: ATTTGATACAGGAGCAGACCTCAAC R: GTGGTGGTTATGGTGGTAAATTTGT	5′UTR	2	0.1166	0.1166	0.1
JM5	(TTA)_22_	F: GCAGAAACTCGGTAGAACTGTGAGT R:GGCATAATCTACTGTTATCTCATCCC	5′UTR	3	0.2998	0.2999	0.2
JM6	(AT)_24_	F: CCAATCGGAGAGTGAAATAGAACAT R: TCTCGAGTTTAAATCTTGGGTATGC	Intron	6	0.2149	0.2149	0.2
JM7	(TA)_24_	F: TGAGAGTGTTACAGAGAGTGTTGCTT R: TGTTACTGCTGAAACATGGAATGAC	Intron	4	0.1499	0.1499	0.1
JM8	(TC)_23_	F: GAATTTAGAAGCCACATTTGAGACG R: CCTATGTAACCCAAGAAAGACGATG	Intron	3	0.1499	0.1499	0.1
JM9	(TC)_15_	F: GTTAGAGAAGGCCAAATTGAAACTG R: ACTTCATTACGTCGAGAGATATCGG	Exon	2	0.1832	0.1833	0.2
JM10	(GAA)_15_	F: TGGAAGACGAATACTATGACGATGA R: CAGGTGCTACTTCTTCTTCTTCAGG	Exon	2	0.1149	0.115	0.1
JM11	(CAT)_12_	F: GCATGCAAACCCTGAATTATGTACT R: GCTGCTGACTCTGTTTCTCCTTCTA	Exon	2	0.2149	0.2149	0.2
JM12	(TA)_33_	F: TTGGCTCATAATAACTCCTCAAAGC R: GCGAGTGCTGTCTAAAGCCTAATTT	Non genic	2	0.1149	0.115	0.1
JM13	(AT)_25_	F: GTCAGTACCTACAAGCTGCCTTCAT R: GCCTTTGGATGAACCTATTCACATA	Non genic	5	0.1832	0.1833	0.2
JM14	(AT)_34_	F: GTTTGGCGATGAGCTAATTGAGATT R: GGCTCGAACCTTTCTGATCTAATGT	Non genic	2	0.2732	0.2732	0.2
JM15	(TA)_42_	F: TGTAGATAGCCTTAGCTGTGCATTG R: GTACTCTCGAGGGAGTTGATTGTGT	Non genic	2	0.5397	0.5398	0.5
JM16	(TA)_38_	F: TTGGCTCATAATAACTCCTCAAAGC R: GCGAGTGCTGTCTAAAGCCTAATTT	Non genic	2	0.4481	0.4481	0.4

JM: *Jatropha* microsatellite; F: forward; R: reverse.

**Table 2:** SSRs used to detect polymorphisms in *Jatropha* species.

SSR locus	Repeat motif	Primer sequence	Location of repeat motif	No. of alleles detected	HO	HE	PIC
JM8	(TC)_23_	F: GAATTTAGAAGCCACATTTGAGACG R: CCTATGTAACCCAAGAAAGACGATG	Intron	3	0.7654	0.7654	0.7
JM10	(GAA)_15_	F: TGGAAGACGAATACTATGACGATGA R: CAGGTGCTACTTCTTCTTCTTCAGG	Exon	2	0.5925	0.5926	0.5
JM11	(CAT)_12_	F: GCATGCAAACCCTGAATTATGTACT R: GCTGCTGACTCTGTTTCTCCTTCTA	Exon	2	0.5679	0.5679	0.5
JM15	(TA)_42_	F: TGTAGATAGCCTTAGCTGTGCATTG R: GTACTCTCGAGGGAGTTGATTGTGT	Non genic	2	0.4938	0.4938	0.4
JM16	(TA)_38_	F: TTGGCTCATAATAACTCCTCAAAGC R: GCGAGTGCTGTCTAAAGCCTAATTT	Non genic	2	0.6913	0.6914	0.6
JM17	(TA)_31_	F: CTTCTCAGCAACATCACATCAAACT R: CGCTAAGTTACATAGGACAAAGGGA	5′UTR	3	0.6172	0.6173	0.6
JM18	(AT)_25_	F: ATTCAGGCCATCCACATAGTCTAAC R: GACCCTATTGATTGATTTAAGAGCC	5′UTR	2	0.5925	0.5926	0.5
JM19	(TA)_33_	F: GCCTTAGTTGTGCATTGCTCTATTT R: ACTCAAACTTATGTCCCAATCGTCT	5′UTR	2	0.7407	0.7407	0.7
JM20	(TA)_23_	F: AGATTTAGAAATGGTAATAGGGCGG R: GACCTATCCGTGTCGTGTAGATTT	5′UTR	7	0.6419	0.642	0.6
JM21	(AT)_25_	F: GCAAGAAATAAGGTACAACCGAAAC R: GTGAGCAATTACCAAAGGAAACAAG	5′UTR	2	0.6913	0.6914	0.6
JM22	(AT)_29_	F: ATGCTATCGGAATAGATCCTTCGAG R: TGGTAAACAAGAGTTGAGGGTTAGG	5′UTR	3	0.4444	0.4444	0.3
JM23	(AT)_32_	F: GAGATGGAAATGATTGGTGTTGAGT R: CGCCTCATCCTCACATTATACACTT	5′UTR	3	0.7654	0.7654	0.7
JM24	(AT)_20_	F: TGATGGATTGAGAACTGAAGAGGAT R: ACTCTAATTAGGCCCAGATTCCAAC	5′UTR	4	0.6913	0.6914	0.6
JM25	(CT)_20_	F: CTGACATATCTTATTGGGTGTGGAA R: TGTAAGAGTATCATCCATTTGCCAG	5′UTR	2	0.6913	0.6914	0.6
JM26	(TC)_18_	F: GCCTTTAAGAGATTGATGGCAACTA R: AAGTATTCATATGCCCTAAGCCTCC	3′UTR	2	0.7160	0.716	0.7
JM27	(TA)_24_	F: TTGGAGGTTACAATCAATGGCA R: GCATGTGCCCGAATTGAATA	3′UTR	3	0.7654	0.7654	0.7
JM28	(AT)_27_	F: CCATTTGGTGTTAATCACATGAGTC R: GACAATAGTGATGTTGGATTCCACT	3′UTR	2	0.7160	0.716	0.7
JM29	(AT)_25_	F: CTGTTTGAGGATCAGACTTTGAAGC R:AAAGAAATAATGAGGAGGGAGGTTG	Intron	2	0.7160	0.716	0.7
JM30	(AT)_25_	F: GCATGGAAATTCAATTCTGTGCTAC R: TTTGGTGATGAGGATTGTTGCT	Intron	2	0.7160	0.716	0.7
JM31	(ATA)_19_	F: TGCTCCCAGTAAGCATAAGAAGAAG R: CTTGCTCGGTTACCATTACCATTAC	Intron	2	0.7160	0.716	0.7
JM32	(AT)_22_	F: AGCAAGAAACCATACTTCGAGTGTC R: GAGATGCCAACCTTTGTGATTAGTT	Intron	2	0.6666	0.6667	0.6
JM33	(GA)_21_	F: TTCAATAACAGATTTGGCTAGGCTC R: GACAATTGAAAGGTGCAATCCTAGT	Intron	3	0.4938	0.4938	0.4
JM34	(AT)_24_	F: CAGACCCATCTGATCATCATTGTAG R: TCCTCAGGTAAATTGCTCATCTTTC	Intron	3	0.5679	0.5679	0.5
JM35	(CT)_12_	F: CAGCGTCCCTCTCTCTCTTCC R: AGGAAGTTGAGGGACCAAATTGTA	Exon	2	0.6419	0.642	0.6
JM36	(AT)_29_	F: GAAAGCTAGAAATCAATGAACGCAC R: TCATTTAGTACATTGACCGGAGACA	Non genic	3	0.6419	0.642	0.6
JM37	(AAT)_16_	F: AATCACATCAGTTGTAACGGCA R: ATAATCTGATGGTTCAGTCAGCTCC	Non genic	3	0.7407	0.7407	0.7

**Table 3:** Extent of amplification and polymorphism among SSRs of varying repeat units in *J. curcas. *

Repeat units	SSRs tested (%)	SSRs with amplification (%)^1^	Polymorphic SSRs (%)^2^
10–15	11	3.00	1.89
16–20	13	12.26	3.31
21–25	14	18.20	6.16
26–30	26	20.20	3.31
31–35	22	9.00	1.89
36–40	8	3.20	0.47
>40	6	4.00	0.47

^1^Percent amplification was calculated as: SSRs amplified from the corresponding repeat unit X100/total no. of SSRs tested for amplification from the corresponding repeat unit.

^2^Percent polymorphism was calculated as: SSRs polymorphic from the corresponding repeat unit X100/total no. of SSRs amplified from the corresponding repeat unit.
