# Supplementary material for: Repertoire of SSRs in the Castor Bean Genome and Their Utilization in Genetic Diversity Analysis in Jatropha curcas
Source: Comp Funct Genomics. 2011 May 22;2011:286089. doi: 10.1155/2011/286089 (PMC3115337; doi:10.1155/2011/286089)
Supplement: Supplementary file 1 — Supplementary materials contains the list of J. curcas genotypes which were used in the present study which were procured from the National Bureau of Plant Genetic Resources (NBPGR), New Delhi, India and a list of additional primer pairs from Castor bean harboring SSRs which can be utilized for transferability and polymorphism survey in Jatropha. [file 286089.f1.pdf]

Supplementary Materials

Table S1: List of genotypes used in diversity analysis

S.No.	Genotypes
1	Urli-Kanchan
2	KcJK5
3	KcPBS19
4	KcPBS15
5	KcJK2
6	PBS SKS25
7	KcPBS16
8	KcPBS22
9	KcPBS26
10	KcJK8
11	KcJK1
12	KcJK6
13	KcJK9
14	KcJK3
15	KcPBS4
16	KcJK10
17	PBS SKS21
18	PBS SKS20
19	Chaterpatti
20	PBS SKS22
21	KcJK11
22	KcPBS2
23	FRI-EL-C5
24	FRI-EL-C2
25	PBS SKS24
26	PBS SKS26
27	KcJk7
28	CSNCRI-Guj-C3
29	SKN-Big
30	CSNCRI-Guj-C2
31	KcPBS17
32	Hissar local
33	SKNJ2
34	KcPBS13
35	PBS SKS27
36	CSNCRI-Guj-C5
37	PBS SKS19
38	CSNCRI-Guj-C4
39	CSNCRI-Guj-C1

40	KcPBS3
41	KcJk4
42	FRI-EL-C4
43	PBS SKS23
44	Hansraj
45	PBS SKS18
46	FRI-EL-C3
47	FRI-EL-C1
48	PBS SKS28
49	KcPBS10

Table S2: SSRs, which can be synthesized and tested on Jatropha as well as castor bean to detect polymorphisms

SSR locus	SSR motif	SSRs position corresponding to gene	Primer F	Primer R
JM38	(AT)21	5'UTR	TTAGATTCACTGCTTTAGGCTTGCT	TTGAACTTCTTACTCATTGCTCGTG
JM39	(TA)22	5'UTR	ACTCAACACCAATCATTTCCATCTC	GAAGAGGAGAGCACCCAGTGTT
JM40	(TTA)23	5'UTR	AGTTCGACTAGTTCCACCAGAATTG	ATTGCCTTCCTCGTCCTAGAGTAAT
JM41	(AT)22	5'UTR	TAGCAACAACTCTATTTAAGCTCAC	AGCGTTAGAATGTTGACTCATCCA
JM42	(TA)22	5'UTR	TTTGAGTGCTCCTATTTGGCTAGAA	CAAATGACAAGTAGGCAGAACTTT
JM43	(ATA)22	5'UTR	TCATAATCGGTCTATTCTTCGTGCT	TTGGTCGTTGTATGGTTAGTCAAAG
JM44	(TA)25	5'UTR	CATTTGTGTGTGCCCATTTCATTAG	CATTATGGATTAGAAGCATGTCACC
JM45	(TA)22	5'UTR	GAGGTACAAGAGAGAAAGCCAACA	CATAGACGCATGTAAATGAGATGG
JM46	(TA)23	5'UTR	GATTTAGAAATGGTAATAGGGCGG	GACCTATCCGTGTCGTGTAGATTT
JM47	(AT)25	5'UTR	ATTCAGGCCATCCACATAGTCTAAC	GACCCTATTGATTGATTTAAGAGCC
JM48	(TTA)22	5'UTR	CAGAAACTCGGTAGAACTGTGAGT	GCATAATCTACTGTTATCTCATCCC
JM49	(AG)24	5'UTR	TTCTCCAAAGCCAGAGATAGACATT	ACCTCTCAACAGGATTTGAACACTC
JM50	(AT)24	5'UTR	AAGATCGATACTTATAAGGTTTGCC	TGGCCGCATATCTTAGTCATTCTA
JM51	(TA)23	5'UTR	GCAACTGAACATAACCAGCCATAA	CATACCCATTGTTGCCCTTGATATT
JM52	(TA)23	5'UTR	AATTAACAGTACTGCCACATGGTCC	CCAACCGTTACCCATTTGATATAGT
JM53	(AT)25	intron	GTACATGAATGAATCGAATCGCAC	TGTGTTATTGGAGATGGTGATGGT
JM54	(TA)22	intron	GCCTCATCCTCACATTACACACTTT	CTTGGAGCATTAGTTAAACCAAACG
JM55	(TA)22	intron	GAGGCACGACAAATTAAGAATAGA	GAGCCAAAGATTGTGGTTATAATGG
JM56	(AT)24	intron	CAGACCCATCTGATCATCATTGTAG	TCCTCAGGTAAATTGCTCATCTTTC
JM57	(TC)23	intron	AAATTACTTCCAGAGACGAATGCCT	CTCACCAACAGGGTAATTTACATGC
JM58	(AT)22	intron	CTTGTTGGAGGGAAGAGAAATAAT	TTTACATCATCTAGCGTGCCATGTT
JM59	(TC)23	intron	AATTTAGAAGCCACATTTGAGACG	CTATGTAACCCAAGAAAGACGATG
JM60	(GA)22	intron	GGATCATCTATGCTTGTAGCGAACT	TTAACAGAGTGAAGGATTTGCCTGT
JM61	(TA)22	Non genic	CAAGTCACACTATACGTCAATCCAA	TACCCGATAAGTACCAATACGCAA

JM62	(TC)24	Non genic	GCATTGATATTGAACACTGGACATC	TAGACATTAGCACAGGCTCAGGAT
JM63	(TC)23	Non genic	ATCAGAAGCCCAATACATCAAAGA G	GATTTCCTACATATAGCCAATGGGA
JM64	(AT)21	Non genic	TAGCAAACTATACACAGGGTTTGG C	AACGCGCACGTTGATAAATAGAAT
JM65	(TA)22	Non genic	CAAGTCACACTATACGTCAATCCAA	TACCCGATAAGTACCAATACGCAA
JM66	(AT)25	Non genic	CGTGCCTAAATCCTGTCACTATCTT	CACACACACACACACACACACATA
JM67	(TC)24	Non genic	GCATTGATATTGAACACTGGACATC	TAGACATTAGCACAGGCTCAGGAT
JM68	(TC)23	Non genic	ATCAGAAGCCCAATACATCAAAGA G	GATTTCCTACATATAGCCAATGGGA
JM69	(TTA)23	Non genic	GCCACAATAATCACAAGGGATAAA C	GTAGCTTCCATCAATCCAGAAGAAA
JM70	(AT)24	Non genic	GCAATGTGGGAGAAATACAAACTT C	GGTAGGCATTCATGCCAGTCTAATA
JM71	(TA)22	Non genic	ACTCAACACCAATCATTTCCATCTC	GCATTGGGTATTGTACCCTCTCTTA
JM72	(GT)21	Non genic	ACTATGGGAGACCATACAGGGAAA	ATAAGGCGAGCTTGTGTATCAACAT
JM73	(TA)24	Non genic	GGGTGTATTCAATTTGCGAGTAAAG	TGTTGTGTGTGTGTCTCTCTCTCTC
JM74	(TA)25	Non genic	TAGCTATTCTCATCATTCCTCCACC	GTCAAAGGTACGCTTCTTGACCTAA
JM75	(AT)24	Non genic	ATGTTCCCTTTCTACGTCTCTTCCT	TCTTCTCTCTAACGCACTCCTGAAT
JM76	(AT)24	Non genic	TCACTTGTTGGGATCTTCTTTAACC	AAAGTCGCCAATTCAGTAATCAGAC
JM77	(AT)24	Non genic	GATGACATTATTGCCTTCTATTGGG	AGCTACTCCCTCCTCTCAAATCCTA
JM78	(TA)24	Non genic	GATGTGATTCGACTTTATGTCGTTG	ACAAGCGTACTACTACAAACTAGGG
JM79	(AT)17	5'UTR	AACACCAATCATTTCCATCTCAGTC	ATGTAGAGCAAGCCTAAAGCAGTG
JM80	(TA)15	5'UTR	GGAAAGCCCAAGAACTTGGAAA	CATGTATAATTTGAGGGATGGCTTC
JM81	(GA)16	5'UTR	GTTAAGTCAAACGTTGTGTTTGTGG	CTTAGCTCATTCCTTGGAGTTGAAG
JM82	(AG)20	5'UTR	AAGAGGATGACCAATGAAGAAAGA	ATTTAACCTCTCCCTTCCCTTCTCT
JM83	(ATT)18	5'UTR	AGATTACTTAGAGTTGCAGGCATTG	AAGGTTCAAGAGAGTGAAGGTTGA
JM84	(CT)15	5'UTR	TTTCCAAGATATACTCTCGAGGTGC	GAGTGAGTGAGTGAAAGGTGGATA
JM85	(GA)18	5'UTR	CAGATAAACGAAACCCTAAATTCC C	GATACATTCACCTCTTCCCAGCTTA
JM86	(TA)15	5'UTR	AACGAGTGAAGAAGAAACTTTGGA	TCTGTTGAATACCTTCGTTACAGCA
JM87	(TC)15	5'UTR	CTGCCCTAACACCAATAAGTGAATC	ACCTCATGTCTTCTCTGTCACTGGT
JM88	(TA)16	5'UTR	TTGCCAAGTGTAAGCTGATTGA	AGTTATTTGTGCACTGTTGACGTGT
JM89	(AT)19	5'UTR	GCGCCTATCTTGAGAAATATTGTGA	AATGTAAGGAGAAGGTGAAAGCCT
JM90	(TA)16	5'UTR	TTCCATGTCCTAGTTTCTTTCTTGG	GCTTCCATATCTCTCTCTCTTTCCC

JM91	(AT)16	5'UTR	GCTAGCAATTGGCCAAACTAAGAT	CGTTTATCTGTGTGTTACACGTCTG
JM92	(GA)17	5'UTR	CTGCAAGAAGAATAGAGAAACAGG	GTCGTCTCCAGAGAATTAAGGGAG
JM93	(TC)18	5'UTR	GCCTTTAAGAGATTGATGGCAACTA	AAGTATTCATATGCCCTAAGCCTCC
JM94	(AT)18	5'UTR	CATAGTTTCGGCTTAATCAACGAGT	CGGCTTGTGAGTAATAAGTTGGTCT
JM95	(AT)21	5'UTR	AGGGTCAGAAGTACACAAGTGAAT	AATTTCTTCTGCAGGCTAAATGGTC
JM96	(CT)17	5'UTR	TTTAGGTGAAGCAAAGAAAGTGGA	GATGCAACTATTAGACGCAGTTTGA
JM97	(AG)18	5'UTR	AGCACTCAGTCCACTGAAATAAAC C	TTATTATGCTTGTGCTGGCTACTCA
JM98	(GA)20	5'UTR	TGATGGCTCAAGTACAAGTTCTAGG	TTTGTGAGTACACAGAGAGAGAAA
JM99	(TC)17	5'UTR	GAGTCACAACTCATAACCCTTTCGT	GGAGAAGGAGAAGAGTGATAAAGG
JM100	(TA)16	5'UTR	TTGGCCTAATACAACAGTCAACAA G	CGGATTAACGCCATTTAGAATAACC
JM101	(AG)18	5'UTR	TTGTTGAGAGTGAGTAGCATTGGA G	AACTGTCGAATTGCTTAAATCCACC
JM102	(TA)16	5'UTR	ATCATACCATATCTGGCAGGGTAA A	TTTAAAGGAATTGGTAGGGCAAGT
JM103	(CT)22	5'UTR	GCATGCAAACCCTGAATTATGTACT	TGCTGACTCTGTTTCTCCTTCTACTT
JM104	(AT)17	5'UTR	TCTCCCTCCCGGAATAGTGTATTTA	AGTAATATTGGCCCTAAGTCGGAAT
JM105	(TAA)17	5'UTR	AGCTTGGTTTGTTCATAAAGGTCAC	AAAGCACACGAAGCTAATCTTTCAC
JM106	(GA)16	5'UTR	GAGATTAGCCAAAGAAATGTGAAG	AATAACCTGACACTAAAGTGGCTCC
JM107	(AT)16	5'UTR	TTGCAGTCCTATCTAACAAATTAGG	GTTGGACCTTCTTTGAGTTGTCTTC
JM108	(AT)20	5'UTR	ATGTCTCTTCAGCAATCCATTCTTC	GTTTCTTATGGGTCGGTTCTGTATG
JM109	(AG)16	5'UTR	ATGTCTCTTCAGCAATCCATTCTTC	GTTTCTTATGGGTCGGTTCTGTATG
JM110	(AT)16	5'UTR	CGTCCATGTGTATTGTATGAAGATG	TGTCCATTATACAGCTATCCCATGA
JM111	(CT)17	5'UTR	TGAAAGCAAACAAAGAGGTCACTC	ATGATCAGTTGCAGATGAGGTGTTA
JM112	(AT)20	5'UTR	TCATCGAATGGTAGAGAACTAATG G	TTAATTCGGATTCTGAGTCTTGAGG
JM113	(TA)16	5'UTR	CTTTCCTTTGCCATGTACTTTCATC	TTACATTGACATCCAGTGGCTAGAA
JM114	(TA)18	intron	TCCGGAGCTTGACTAGAAGATAGA A	GGCAGTTTCAGAGAGTCTTACAACC
JM115	(AT)16	intron	CTCTCTGAAGGTTTAACACAACGCT	CATAAGCGCTTTAATTTAGGACTGC
JM116	(TA)17	intron	CTGTAGTGGACTGCTGTCACTTGTT	GCACTTAGAAAGACTCTTAGCCAGC
JM117	(CT)18	intron	CATAGCATAGCATAGCAAGTGGAT G	GAAGGGATACTGCAGACATTAGAA
JM118	(TA)17	intron	TTCTCTAGATTATGCACAGCACAGC	ACATGGTGGACATTATCACTCACAA
JM119	(CT)17	intron	TCAACTTCTACTTCAAACACGCATC	CATACTTGGTCCTGATGTACTGTGA
JM120	(TA)17	intron	AAACACGCATCTATTACACCATCCT	CATACTTGGTCCTGATGTACTGTGA
JM121	(TA)18	intron	AGATAACGAGATCGATGACAACAC	AGGGAGAATATTGGTTTCAAAGGT
JM122	(ATT)16	intron	AAACGGGTCCCAAACCTATCTC	GGTTTGATGCCTAAATTGTGAGGT
JM123	(CT)17	intron	TCAACTTCTACTTCAAACACGCATC	CATACTTGGTCCTGATGTACTGTGA
JM124	(TA)17	intron	AAACACGCATCTATTACACCATCCT	CATACTTGGTCCTGATGTACTGTGA
JM125	(TA)20	intron	GAATATGGACTAACGTGGTTTGCTT	ATTGCTGGAACTTAGCAGTCAATTC

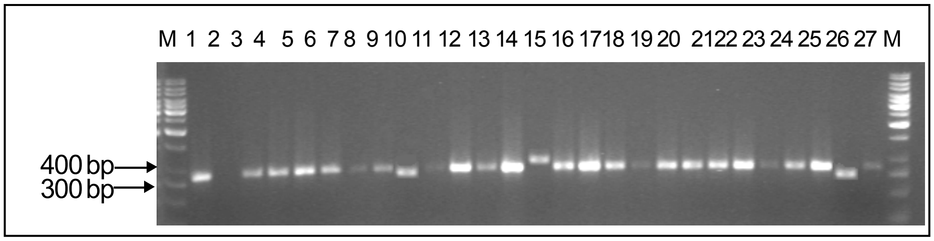

Figure S1: Polymorphisms in amplicon size for intronic SSR (TC)23 at locus JM8 in Jatropha genotypes (1-27)

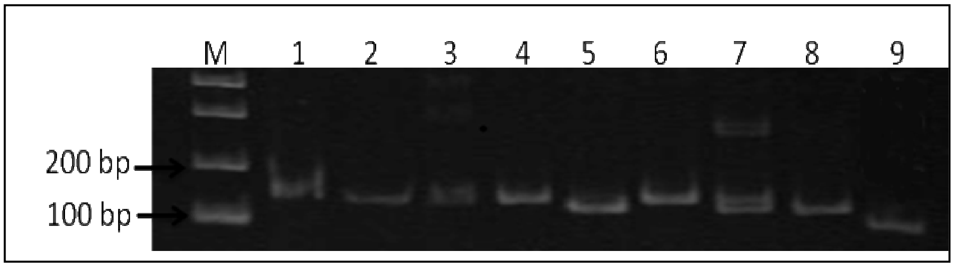

Figure S2: M =Molecular weight marker; Polymorphism at SSR locus JM22 (5'UTR) (AT)29 on Jatropha species (1- *J.maheshwarii*, 2- *J.multifida*, 3- *J.gossypifolia*, 4- *J.podagrica*, 5- *J.glandulifera*, 6- *J.curcas,* 7- *J.tanjorensis,* 8- *J.villosa,* 9- *J.integerrima*
